# Detection of specific antibodies against *Leishmania infantum* in canine serum and oral transudate using an in-house ELISA

**DOI:** 10.1186/s13071-022-05246-2

**Published:** 2022-05-10

**Authors:** Marta Baxarias, Júlia Viñals, Alejandra Álvarez-Fernández, Mª Magdalena Alcover, Laia Solano-Gallego

**Affiliations:** 1 Departament de Medicina i Cirurgia Animals, Facultat de Veterinària, Universitat Autònoma de Barcelona, Cerdanyola del Vallès, Barcelona Spain; 2 Departament de Biologia, Sanitat i Medi Ambient, Facultat de Farmàcia i Ciències de l’Alimentació, Universitat de Barcelona, Barcelona, Barcelona Spain

**Keywords:** Leishmaniosis, Dog, Oral transudate, Serology, Diagnosis, Spain

## Abstract

**Background:**

Canine leishmaniosis caused by the protozoan *Leishmania infantum* is a complex infection due to its variable clinical signs and laboratory findings. Therefore, a broad range of techniques is available for diagnosis. Testing for specific antibodies in serum is the most commonly used technique, although the testing of other body fluids, such as oral transudate (OT), can be an alternative as its collection is non-invasive and testing can be performed by untrained personnel. The aim of this study was to assess and compare the detection of *L. infantum*-specific antibodies in paired samples of serum and OT collected from apparently healthy dogs and dogs with clinical leishmaniosis using an in-house enyzme-linked immunosorbent assay (ELISA).

**Methods:**

Serum and OT were collected from 407 dogs, which varied in breed, sex, age, lifestyle and clinical status, by many practicing veterinarians in Spain. The main geographical areas of sampling included Barcelona (*n* = 110), Mallorca (*n* = 94), Cadiz (*n* = 54) and Asturias (*n* = 47). The majority of infected dogs were apparently healthy (89.9%) while 41 presented clinical signs and/or clinicopathological abnormalities compatible with *L. infantum* infection and subsequently diagnosed with leishmaniosis (10.1%). An in-house ELISA was performed to quantify the anti-*Leishmania* antibodies in serum and OT.

**Results:**

The *L. infantum* infection rate determined by the in-house ELISA was 37.1% in serum samples and 32.7% in OT samples. Serum and OT ELISA results showed a positive correlation (Spearman's correlation coefficient *r*_s_ = 0.6687, *P* < 0.0001). The percent agreement between the serum and OT ELISA results was 84%, while agreement according to Cohen's kappa statistic (*κ*) was substantial (0.66) when all samples were analyzed. The highest percent agreement (92.1%) between both tests was found in dogs from low endemicity regions and from sick dogs, with both groups presenting almost perfect agreement according to Cohen’s *κ* agreement test (0.84). Few seronegative dogs (*n* = 23) tested positive by the OT ELISA. The agreement between serum and OT went from almost perfect to moderate when the geographical distribution and clinical status were analyzed.

**Conclusions:**

The results of this study demonstrated an almost perfect to moderate agreement between OT and serum samples tested using the in-house ELISA. These results are particularly promising in sick dogs with high antibody levels while the results seem less optimal in apparently healthy dogs with low antibody levels.

**Graphical Abstract:**

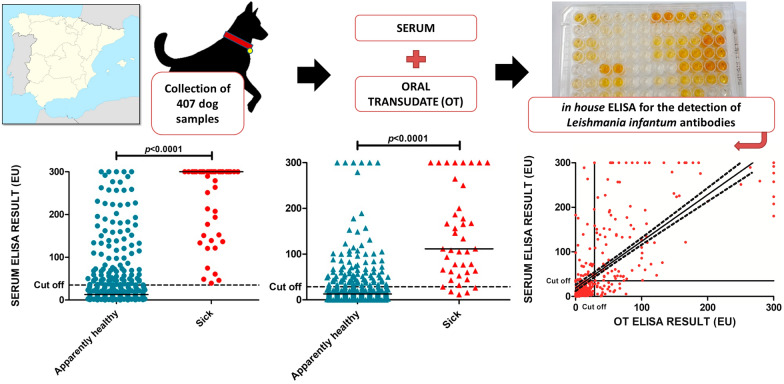

## Background

Canine leishmaniosis (CanL), a zoonotic and endemic protozoan disease caused by *Leishmania infantum*, is endemic in the Mediterranean basin [1, 2]. Transmission is mostly through the bite of a female phlebotomine sand fly following a digenetic life-cycle which consists of two different phases: an extracellular and mobile promastigote in the sand fly, and an intracellular and non-motile amastigote in the mammalian host [3]. Other confirmed transmission routes, such as venereal [4, 5] and transplacental [5, 6] transmission and through blood transfusion, also occur [7, 8]. The dog is considered to be the main domestic reservoir for *L. infantum* infection in the Mediterranean basin [2, 9], while other mammals may be able to maintain a wild-life cycle [10–12].

The seroprevalence of *L. infantum*-infected healthy dogs in western Europe was 23% between 1971 and 2006 [13]. In Spain, the seroprevalence has been reported to be around 10%, although it can vary from 0 to 57% depending on the region [14]. Moreover, the prevalence of dogs that develop the clinical disease is usually lower than 10% [15, 16]. CanL is a complex infection due to its variable clinical manifestations and wide spectrum of clinical signs and laboratory findings [9, 17, 18]. One factor underlying this variability is the dog’s immune response, which requires a balance between inflammatory and regulatory responses to control *L. infantum* infection [19]. For example, neutrophils and macrophages play distinctive roles in the dog’s initial immune ability to control the infection or to allow progression towards disease. Both neutrophils and macrophages phagocytize the parasite which can lead either to the elimination of the parasite through the production of reactive oxygen species (ROS), or to the survival of parasites within macrophages, leading to parasite persistence and dissemination [19]. T lymphocytes also play an integral role in preventing parasite growth and disease development as these T cells produce interferon gamma (IFN-γ) among other cytokines, such as tumor necrosis factor alpha, interleukin-2 or chemokines, which results in the differentiation, recruitment and activation of macrophages. However, as the infection progresses towards disease, there is a decrease in T cell proliferation and IFN-γ production and a lack of macrophage activation, resulting in a reduction of parasite elimination [19]. Many other factors can also affect the development of the disease, such as age, sex, host genetics, among others. To date, however, the mechanisms responsible for the dog’s resistance or susceptibility are still unknown [15, 17].

Due to this complexity, CanL diagnosis often requires an integrated approach, including a clinicopathological examination and specific laboratory tests [9, 15, 18]. A full clinical history, thorough physical examination and several routine diagnostic tests, such as a complete blood count, biochemical profile, urinalysis and serum electrophoresis, are necessary when there is a suspicion of CanL [15, 18]. In addition, several diagnostic techniques are available that enable a definitive diagnosis of *L. infantum* infection, such as parasitological diagnosis (direct observation of the parasite), serological techniques (such as the enzyme-linked immunosorbent assay [ELISA] and indirect fluorescent antibody test) and molecular studies (such as quantitative PCR) [1, 17, 18, 20]. Parasitological methods and molecular studies can detect the presence of the parasite, by direct observation or detection of DNA, respectively, while serological techniques detect serum anti-*Leishmania* antibodies. The diagnostic techniques must be used with full knowledge of the basis of each test and its limitations, as well as how to correctly interpret the results [15, 17, 18].

Interestingly, these diagnostic techniques can be performed using different types of samples, such as blood, serum, urine and other infected tissues [15, 21–23]. The use of alternative samples, such as oral transudate (OT), hair or conjunctival swabs, has also been studied, with interesting results [24–27]. Immunoglobin A (IgA) can be found in OT as it is secreted in the salivary glands by plasma cells, along with immunoglobin G (IgG) and immunoglobin M (IgM), both of which are derived from plasma [28]. Specific antibodies against *L. infantum* have been previously detected in saliva samples of infected sick dogs only by means of a time-resolved immunofluorometric assay (TR-IFMA) [24, 29–31]. However, to the authors’ best knowledge, the detection of antibodies against *L. infantum* by ELISA in OT from apparently healthy dogs has not been previously documented. The advantages of using OT instead of serum include a non-invasive, cheap and painless collection of the sample, which can also be performed by untrained personnel.

The aim of this study was to assess and compare the detection of *L. infantum*-specific antibodies in paired samples of serum and OT from apparently healthy dogs and from dogs with clinical leishmaniosis, using an in-house ELISA.

## Methods

### Dogs

**Fig. 1:**
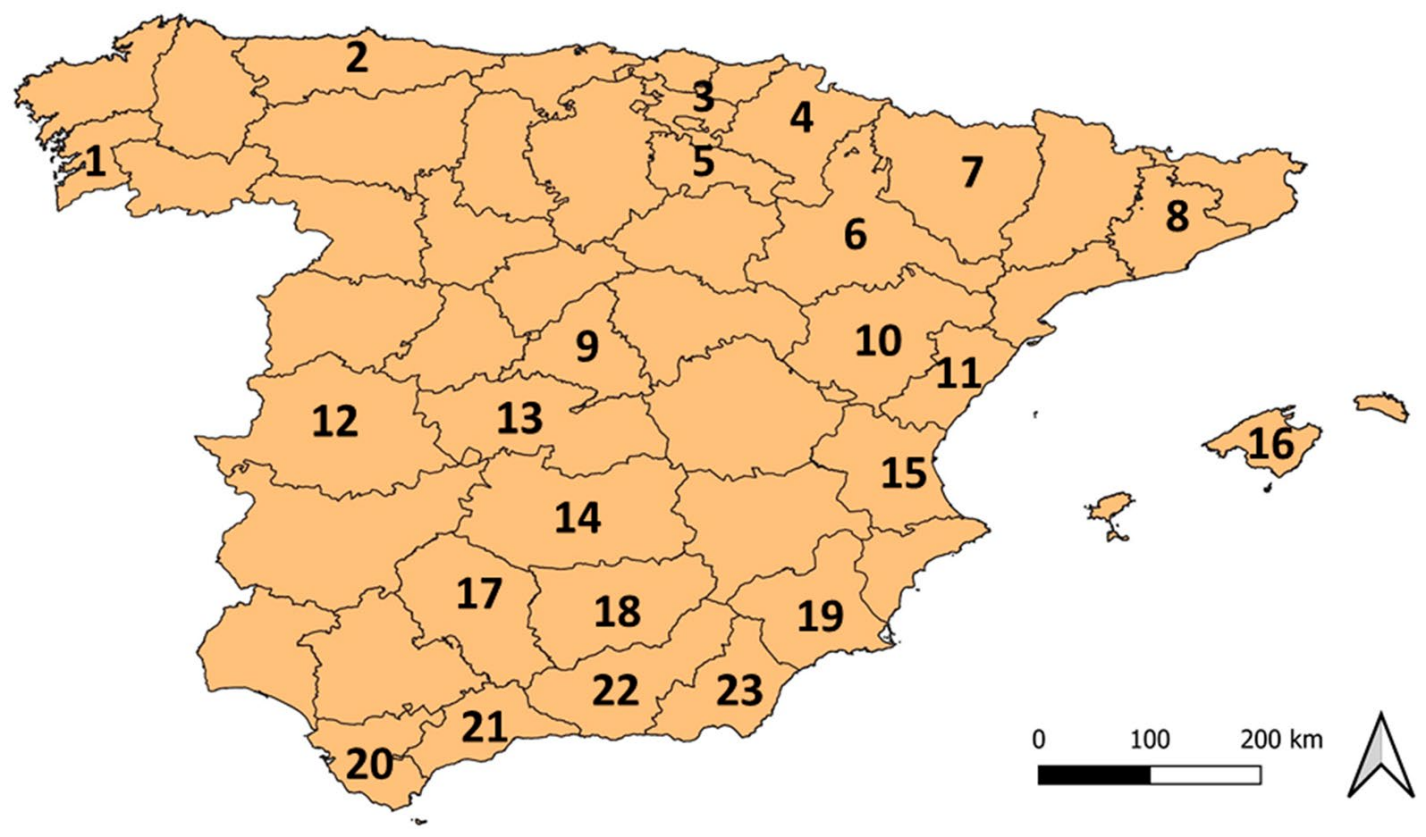
Geographical distribution of dogs sampled in Spain:* 1* Pontevedra (*n* = 5),* 2* Asturias (*n* = 47),* 3* Álava (*n* = 3),* 4* Navarra (*n* = 3),* 5* La Rioja (*n* = 1),* 6 *Zaragoza (*n* = 10),* 7* Huesca (*n* = 1),* 8* Barcelona (*n* = 110),* 9* Madrid (*n* = 8),* 10* Teruel (*n* = 3),* 11* Castellón (*n* = 19),* 12* Cáceres (*n* = 3),* 13* Toledo (*n* = 1),* 14* Ciudad Real (*n* = 6),* 15* Valencia (*n* = 15),* 16* Mallorca (*n* = 94),* 17* Córdoba (*n* = 6),* 18* Jaén (*n* = 2),* 19* Murcia (*n* = 10),* 20* Cádiz (*n* = 54),* 21* Málaga (*n* = 4),* 22* Granada (*n* = 1),* 22* Almería (*n* = 1)

A minimum sample size of 310 dogs was calculated [32] using an expected seroprevalence of *L. infantum* infection of 10% [14] and a power of 80%. Both serum and OT samples from 407 dogs varying in breed, sex, age, lifestyle and clinical status were collected between January of 2018 and June of 2021 by several veterinarians practicing in different areas of Spain (Fig. 1), a country endemic for CanL [14]. Dogs were chosen randomly from veterinary clinics, dog shelters and groups of hunting dogs. The clinical data recorded included the signalment and clinical status of all dogs (Table 1). None of the dogs were vaccinated against CanL. Dogs were considered young if they were aged ≤ 1.5 years, while dogs aged > 1.5 years were considered to be adult. Dog characteristics, such as sex, age, breed and clinical status, and the significant differences between dogs are shown in Table 1.

**Table 1:** Signalment and geographical distribution of dogs enrolled in the study

Geographical distribution (number of dogs)	Sex (%, number of dogs)		Breed (%, number of dogs)		Most common breeds (%, number of dogs)	Age (%, number of dogs)^a^		Age, median (years, min–max)^a, e^	Clinical status (%, number of dogs)	
	Female^b^	Male^b^	Purebred^c^	Crossbreed^c^		Young^d^	Adult^d^		Healthy^f^	Sick^f^
Asturias (47)	51.1%, 24	48.9%, 23	89.4%, 42	10.6%, 5	English setter (17%, 8) and Mastiff (10.6%, 5)	8.9%, 4	91.1%, 41	5.5%, 0.5–12	100%, 47	0%, 0
Barcelona (110)	46.4%, 51	53.6%, 59	20.9%, 23	79.1%, 87	German Shepherd (4.5%, 5) and Labrador Retriever (3.6%, 4)	25.5%, 24	74.5%, 70	4%, 0.3–12	87.3%, 96	12.7%, 14
Cádiz (54)	44.4%, 24	55.6%, 30	29.6%, 16	70.4%, 38	Spanish sighthound (11.1%, 6)	17.5%, 7	82.5%, 33	3.5%, 0.5–16	100%, 54	0%, 0
Mallorca (94)	68.1%, 64	31.9%, 30	80.9%, 18	19.1%, 76	Ibizan Hound (54.3%, 51), Mallorca Shepherd dog (5.3%, 5) and Andalusian wine-cellar rat-hunting dog (5.3%, 5)	38%, 35	62%, 57	3%, 0.5–14	92.6%, 87	7.4%, 7
Total of provinces of origin (407)	51.4%, 209	48.6%, 198	46.7%, 190	53.3%, 217	Ibizan Hound (12.8%, 52), German Shepherd (3.9%, 16) and Mastiff (3.4%, 14)	22.8%, 79	77.2%, 267	4%, 0.3–16	89.9%, 366	10.1%, 41

*max* maximum, *min* minimum

^a^Age was not recorded in 2 dogs from Asturias, 16 dogs from Barcelona, 14 dogs from Cádiz, 2 dogs from Mallorca and 27 dogs from other Spanish regions

^b^Mallorca had a higher rate of female dogs (Chi-square test: *X*^2^ = 11.7, *df* = 3, *P* = 0.008)

^c^Asturias and Mallorca had a higher rate of purebred dogs (Chi-square test: *X*^2^ = 110.9, *df* = 3, *P* < 0.001)

^d^Mallorca had significantly more young dogs than Asturias (Fisher’s exact test: *P* < 0.0001) and Cádiz (Fisher’s exact test: *P* = 0.025), while Asturias had significantly more adult dogs than Barcelona (Fisher’s exact test: *P* = 0.024)

^e^Dogs from Mallorca were significantly younger than those from Asturias (Mann–Whitney test: *U* = 2740, *n*_1_ = 45, *n*_2_ = 92, *P* = 0.002) and Barcelona (Mann–Whitney test: *U* = 5106, *n*_1_ = 94, *n*_2_ = 92, *P* = 0.032)

^f^Barcelona and Mallorca had some sick dogs (Chi-square test: *X*^2^ = 13.4, *df* = 3, *P* = 0.004) while all dogs in Asturias and Cádiz were apparently healthy

The main sampling areas included Barcelona (*n* = 110 dogs), Mallorca (*n* = 94), Cádiz (*n* = 54) and Asturias (*n* = 47) (Table 1). In the additional sampling areas, fewer than 20 dogs were sampled per area, with a total of 102 dogs (Fig. 1). Dogs were also classified according to their clinical status. The majority of dogs were apparently healthy (89.9%) while 41 presented clinical signs and/or clinicopathological abnormalities compatible with *L. infantum* infection and were diagnosed with leishmaniosis (10.1%) [9] (Table 1). Most dogs were sampled at the time of diagnosis and had not previously been treated with anti-*Leishmania* drugs, with the exception of three dogs that had been recently treated with allopurinol. Dogs from Asturias, an area with very low endemicity [14, 33], were classified as negative controls, while samples from sick dogs that were diagnosed with leishmaniosis were classified as positive controls.

### Sampling

Blood samples were obtained by jugular or cephalic venepuncture and later centrifuged (Heraeus Labofuge 400R Centrifuge; Thermo Fisher Scientific, Waltham, MA, USA) at 789 *g* for 10 min to obtain serum.

OTs were collected by foam swabs (Ecouvillon PP; Dominique Dutscher, Bernolsheim, France) impregnated with hypertonic saline (NaCl 7.5%; B. Braun Melsungen AG, Melsungen, Germany) mainly as described previously [34] but with some modifications. The swabs were kept in the dog’s mouth between the gum and the inner mucosa of the upper or lower lip for around 2 min and later centrifuged (Eppendorf Centrifuge 5418; Merck KGaA, Darmstadt, Germany) at 16,000 *g* for 1 min. After that, OTs were collected.

All samples, including both serum samples and OTs, were identified and stored at – 80 °C until further use.

### Quantitative in-house ELISA for the detection of *L. infantum*-specific antibodies

#### Serum ELISA

The in-house ELISA was performed on serum samples of all dogs studied as previously described [21]. Briefly, samples were diluted to 1:800 in phosphate buffered saline (PBS) Tween with 1% dry milk and incubated at 37 °C for 1 h, following which they were washed three times (3 min each wash) with PBS-Tween and once (1 min) with PBS. The samples were then incubated for 1 h at 37 °C with peroxidase-conjugated Protein A (Peroxidase Conjugate Protein A; Merck KGaA) at a concentration of 0.16 ng/µl. After incubation, the plates were washed three times with PBS-Tween followed by an additional wash with PBS. Then, *o*-phenylenediamine and substrate buffer (SIGMAFAST OPD; Merck KGaA) were added to the plates and the reaction was finally stopped with 5 M H_2_SO_4_. The results were read at 492 nm in a spectrophotometer (MB-580 HEALES; Shenzhen Huisong Technology Development Co., Ltd, Shenzhen, China) and were defined as ELISA units (EU) in relation with a positive canine serum sample used as a calibrator set at 100 EU. The cut-off of the serum in-house ELISA was already determined to be 35 EU using the ELISA results of 80 dogs from a non-endemic area, as previously described [35]. Cut-off was established by the standard deviation (SD) method, consisting of multiplying the SD of the results by four and adding up the mean of the results obtained by the ELISA (mean + 4 SD). Serum was classified as high positive when the result was ≥ 300 EU, medium positive when the result was ≥ 150 EU and < 300 EU, low positive when the result was ≥ 35 EU and < 150 EU and negative when the result was < 35 EU [35].

#### Oral transudate ELISA

The in-house ELISA was performed on OTs of all dogs studied as previously described [21] with some modifications. OT samples were diluted to 1:5 in PBS-Tween with 1% dry milk and incubated at 37 °C for 1 h. Washes were performed as described for the serum samples, and peroxidase conjugated Protein A (Peroxidase Conjugate Protein A; Merck KGaA) at a concentration of 0.5 ng/µl was added and then incubated at 37 °C for 1 h. Washes were repeated and *o*-phenylenediamine and substrate buffer (SIGMAFAST OPD; Merck KGaA) were added to the samples. The reaction was stopped with 5 M H_2_SO_4_. As described for the serum samples, the results were read in a spectrophotometer (MB-580 HEALES; Shenzhen Huisong Technology Development Co., Ltd.) at 492 nm and were quantified as EU relative to a positive canine OT sample used as a calibrator set at 100 EU. The cut-off of the OT in-house ELISA was established using the ELISA results of 30 non-infected healthy Beagles. With the values of these 30 dogs, the SD was calculated and multiplied by 4, and then added up to the mean of all the results (mean + 4 SD), resulting in a cut-off value of 28 EU. The OTs were then classified as positive when the result was ≥ than 28 EU and negative when it was < 28 EU.

### Statistical analysis

A descriptive analysis of all collected data was performed. Qualitative variables (sex [female/male], breed [purebred/mixed breed], age [young/adult] and ELISA results [positive/negative]) were assessed with a Fisher’s exact test when only two groups were compared and with a Chi-square test when there were more than two groups. Quantitative variables (age, EU) were assessed using a non-parametric Mann–Whitney U-test when two groups were compared (clinical status: apparently healthy/sick), and the Kruskal–Wallis H-test was used when more than two groups were compared (geographical distribution). Spearman’s correlation test was carried out to detect a relationship between ELISA quantitative results of the serum and OT.

The agreement between the interpretation of the results of serum and OT ELISAs was calculated by percent agreement and by Cohen’s kappa statistic (κ) for agreement (kappa agreement test). When evaluating kappa agreement, the agreement was considered to be slight when it ranged from 0.00 to 0.20, fair when at range 0.21–0.40, moderate at range 0.41–0.60, substantial at range 0.61–0.80 and almost perfect at range 0.81–1.00 [36].

A *P*-value of < 0.05 was considered to be statistically significant. The Shapiro–Wilk test was performed to detect normal distribution of quantitative variables. Areas where < 20 dogs were sampled were excluded from the geographical distribution analysis. The statistical analysis was performed using the package Stats for R software version i386 3.6.1 for Windows. Cohen’s κ statistic for agreement was calculated using free on-line GraphPad software (https://www.graphpad.com/quickcalcs/kappa1/). Graphs were plotted using Graphad Prism version 5.00 for Windows (GraphPad Software, San Diego, CA, USA).

## Results

### Serum ELISA results

The rate of *L. infantum* infection determined by serum ELISA and the serological status of dogs (negative, low positive, medium positive or high positive) are shown in Table 2. The infection rate was significantly higher in adult dogs than in young dogs (42.7 vs 21.5%; Fisher’s exact test: *P* = 0.001), and lower in apparently healthy dogs than in sick dogs (29.5 vs 100%; Fisher’s exact test: *P* < 0.0001) (Table 2). No significant differences were observed between dogs of different sex and breed (Table 2). When dogs from different geographical locations were compared, a significantly lower rate of infection was found in Asturias when compared to the other locations (Chi-square test: *χ*^2^ = 23.7, *df* = 3, *P* < 0.001) (Table 2).

**Table 2:** Rate of *L. infantum* infection, percent agreement and Cohen’s kappa agreement between enzyme-linked immunosorbent assay results for serum and oral transudate samples

Classification (number of dogs)	Number of positive dogs (%)		Percent agreement (%)	Cohen’s* κ* agreement (interpretation)	95% CI of Cohen’s* κ* agreement
	Serum ELISA	OT ELISA			
Total of dogs (407)	149 (36.6)	133 (32.7)	345 (84.8)	0.66 (substantial agreement)	0.59–0.74
Sex					
Female (209)	78 (37.3)	71 (34)	174 (83.3)	0.64 (substantial agreement)	0.53–0.75
Male (198)	71 (35.9)	62 (31.3)	171 (86.4)	0.7 (substantial agreement)	0.59–0.8
*Age*^a^					
Young (79)	17 (21.5)^b^	15 (19)^c^	69 (87.3)	0.61 (substantial agreement)	0.39–0.83
Adult (267)	114 (42.7)^b^	103 (38.6)^c^	224 (83.9)	0.67 (substantial agreement)	0.58–0.76
*Breed*					
Purebred (190)	63 (33.2)	63 (33.2)	158 (83.2)	0.62 (substantial agreement)	0.5–0.74
Mixed breed (217)	86 (39.6)	70 (32.3)	187 (86.2)	0.7 (substantial agreement)	0.6–0.8
*Geographical distribution*					
Asturias (47)	0 (0)^d^	3 (6.4)^e^	44 (93.6)	-^h^	-^h^
Barcelona (110)	30 (27.3)^d^	23 (20.9)^e^	99 (90)	0.73 (substantial agreement)	0.58–0.88
Cádiz (54)	9 (16.7)^d^	7 (13)^e^	48 (88.9)	0.56 (moderate agreement)	0.25–0.87
Mallorca (94)	33 (35.1)^d^	28 (29.8)^e^	74 (79.6)	0.54 (moderate agreement)	0.36–0.72
*Clinical status*					
Sick (41)	41 (100)^f^	37 (90.2)^g^	37 (90.2)	-^h^	-^h^
Apparently healthy (366)	108 (29.5)^f^	96 (26.2)^g^	308 (84.2)	0.61 (substantial agreement)	0.52–0.7
Negative control (Asturias) and positive control dogs (Sick) (88)	41 (46.6)	40 (45.5)	81 (92.1)	0.84 (almost perfect agreement)	0.73–0.95
*Serological status*					
High positive (26)	26 (100)	26 (100)	26 (100)	-^h^	-^h^
Medium positive (40)	40 (100)	34 (85)	34 (85)	-^h^	-^h^
Low positive (83)	83 (100)	50 (60.2)	50 (60.2)	-^h^	-^h^
Negative (258)	0 (0)	23 (8.9)	235 (91.1)	-^h^	-^h^
Negative control (Asturias) and high and medium positive dogs (113)	66 (58.4)	63 (55.8)	104 (92)	0.84 (almost perfect agreement)	0.74–0.94

*CI* confidence interval,* ELISA* enzyme-linked immunosorbent assay, *OT* oral transudate

^a^Age was not recorded in 61 dogs

^b^Fisher’s Exact test: *P* = 0.001

^c^Fisher’s Exact test: *P* = 0.001

^d^Chi-square test: *χ*^2^ = 23.7, *df* = 3, *P* < 0.001

^e^Chi-square test: *χ*^2^ = 12.8, *df* = 3, *P* = 0.004

^f^Fisher’s Exact test: *P* < 0.0001

^g^Fisher’s Exact test: *P* < 0.0001

^h^Cohen’s kappa (*κ*) agreement could not be calculated in the Asturias, the seropositive sick dogs and the serological status groups because of the lack of positivity to both tests or the lack of negativity to both tests

**Fig. 2:**
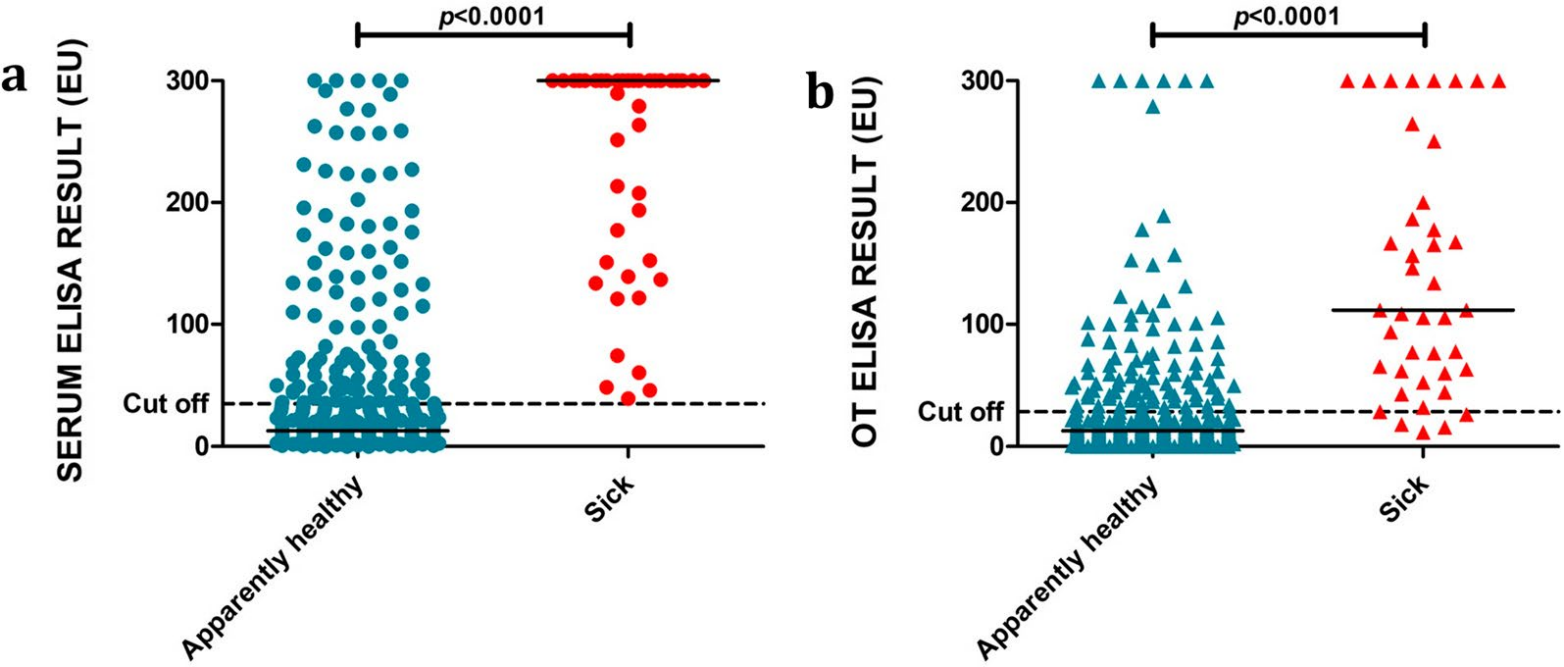
Antibody levels against *L. infantum* (EU) as determined by the in-house ELISA performed on serum (**a**) and OT (**b**) samples collected from dogs classified according to clinical status (apparently healthy vs sick). Horizontal solid black lines indicate the median. Horizontal black dashed lines indicate the cut-off: 35 EU in serum ELISA and 28 EU in OT ELISA.* ELISA* enzyme-linked immunosorbent assay, *EU* ELISA units, *OT* oral transudate

**Fig. 3:**
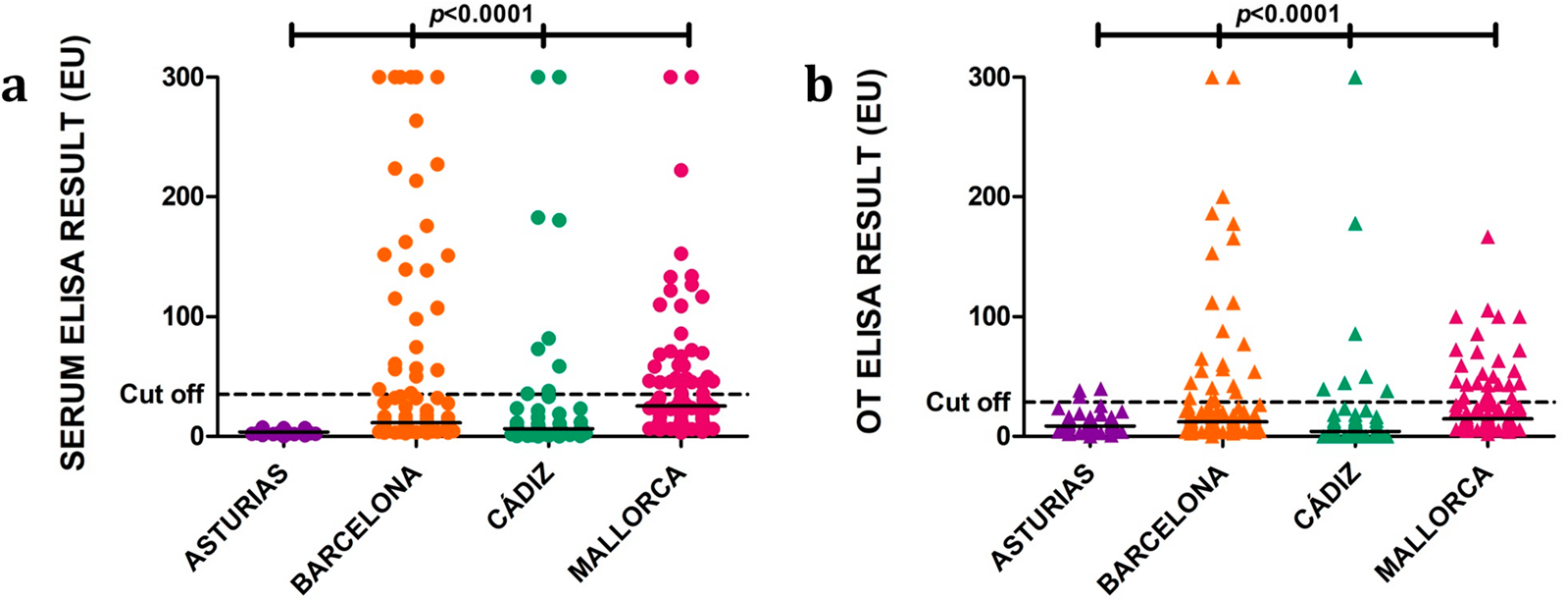
Antibody levels against *L. infantum* (EU) by the in-house ELISA performed on serum (**a**) and OT (**b**) samples collected from dogs classified according to geographical distribution. Horizontal solid black lines indicate the median. Horizontal black dashed lines indicate the cut-off: 35 EU in serum ELISA and 28 EU in OT ELISA.  *ELISA* enzyme-linked immunosorbent assay, *EU *ELISA units, *OT* oral transudate

Regarding the quantitative ELISA results shown in Table 3, adult and sick dogs presented significantly higher median EU values than young and apparently healthy dogs, respectively (Fig. 2; Mann–Whitney test: *U* = 12,389, *n*_1_ = 267, *n*_2_ = 79, *P* = 0.018; Mann–Whitney test: *U* = 829, *n*_1_ = 366, *n*_2_ = 41, *P* < 0.0001). No significant differences were observed when different sexes and breeds were compared (Table 3). When groups from different geographical locations were compared (Table 3; Fig. 3a), Asturias (3.7 EU) presented a significantly lower median value than Barcelona (11.4 EU), Cádiz (6.3 EU) and Mallorca (25.3 EU) (Kruskal–Wallis H-test: *χ*^2^ = 99.2, *df* = 3, *P* < 0.0001) while Barcelona and Mallorca had significantly higher median values than Cádiz.

**Table 3:** Median values of serum and OT EU according to the degree of reactivity to sera ELISA

Classification of dogs (number of dogs)	Median of serum EU (min–max)^a^	Median of OT EU (min–max)^a^
Total of dogs (407)	17.7 (0–300)	14.9 (0–300)
*Sex*		
Female (209)	22.3 (0–300)	13.8 (0–300)
Male (198)	15.9 (0–300)	15.8 (0–300)
*Age*^b^		
Young (79)	11.0 (1.8–300)^c^	9.9 (0–250.5)^f^
Adult (267)	22.3 (0–300)^c^	18.1 (0–300)^f^
*Breed*		
Purebred (190)	16.9 (0–300)	16.0 (0–300)
Mixed breed (217)	18.2 (0–300)	13.6 (0–300)
*Geographical location*		
Asturias (47)	3.7 (0–7.4)^d^	8.6 (0.2–39.9)^g^
Barcelona (110)	11.4 (2.7–300)^d^	12.0 (0.2–300)^g^
Cádiz (54)	6.3 (0–300)^d^	4.1 (0–300)^g^
Mallorca (94)	25.3 (3.2–300)^d^	14.7 (2.2–166.5)^g^
*Clinical status*		
Sick (41)	300.0 (39.3–300)^e^	111.7 (11.6–300)^h^
Apparently healthy (366)	12.8 (0–300)^e^	12.9 (0–300)^h^
*Serological status*		
Negative (258)	7.0 (0–34.7)	9.7 (0–76.4)
Low positive (83)	59.2 (35–142.9)	38.1 (0–166.5)
Medium positive (40)	210.4 (150.4–291.8)	80.4 (0–300)
High positive (26)	300.0 (300)	160.9 (28.5–300)
Total positives (149)	132.8 (35–300)	59.2 (0–300)

*EU* ELISA units, *OT *oral transudate, *max* maximum, *min* minimum

^a^Samples with a value of 300 EU may actually be higher as the spectrophotometer is only able to read up to 3 units of optical density

^b^Age was not recorded in 61 dogs

^c^Mann–Whitney test: *U* = 12,389, *n*_1_ = 267, *n*_2_ = 79, *P* = 0.018

^d^Kruskal–Wallis *H*-test: *χ*^2^ = 99.2, *df* = 3, *P* < 0.0001

^e^Mann–Whitney test: *U* = 829, *n*_1_ = 366, *n*_2_ = 41, *P* < 0.0001

^f^Mann–Whitney test: *U* = 12,863, *n*_1_ = 267, *n*_2_ = 79, *P* = 0.003

^g^Kruskal–Wallis *H*-test: *χ*^2^ = 38.7, *df* = 3, *P* < 0.0001

^h^Mann–Whitney test: *U* = 1461, *n*_1_ = 366, *n*_2_ = 41, *P* < 0.0001

### Oral transudate ELISA results

The rate of *L. infantum* infection determined on OT ELISA is shown in Table 2. Similar to the results for the serum samples, the rate of OT sample positivity was also significantly higher in adult (Fisher’s exact test: *P* = 0.001) and sick dogs (Fisher’s exact test: *P* < 0.0001) (38.6%) when compared to young dogs (19%) while it was lower in apparently healthy dogs than in sick dogs (26.2% vs 90.2%) (Table 2). No significant differences were observed in terms of sex and breed (Table 2). When comparisons were made between groups of dogs from different geographic locations, a significantly lower rate of infection was still found for dogs from Asturias compared to those from other locations (Chi-square test: *χ*^2^ = 12.8, *df* = 3, *P* = 0.004) (Table 2).

Regarding the quantitative ELISA results shown in Table 3, as found in the serum results, adult and sick dogs presented a significantly higher mean EU value than young and apparently healthy dogs, respectively (Mann–Whitney test: *U* = 12,863, *n*_1_ = 267, *n*_2_ = 79, *P* = 0.003; Mann–Whitney test: *U* = 1461, *n*_1_ = 366, *n*_2_ = 41, *P* < 0.0001) (Fig. 2b). No significant differences were observed between different sex and breed (Table 3). When groups of dogs from different geographical location were compared (Table 3; Fig. 3b), Asturias (8.6 EU) and Cádiz (4.1 EU) presented a significantly lower mean EU value than Barcelona (12 EU) and Mallorca (14.7 EU) (Kruskal–Wallis H-test: *χ*^2^ = 38.7, *df* = 3, *P* < 0.0001).

### Correlation and comparison between ELISA results for serum and OT samples

**Fig. 4:**
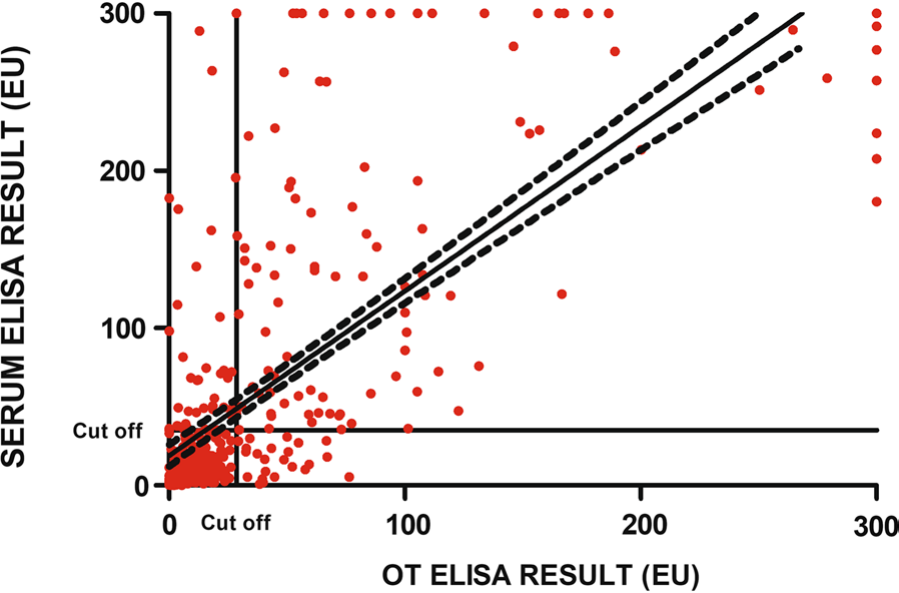
Spearman’s correlation coefficient (*r*_s_) for the serum and OT ELISA results (*r*_s_ [407] = 0.6687, *P* < 0.0001). Red filled circles indicate the individual results for each sampled dog. The horizontal solid black line indicates the cut-off: 35 EU in serum ELISA (*Y*-axis) and 28 EU in OT ELISA (*X-*axis). *ELISA* enzyme-linked immunosorbent assay, *EU* ELISA units, *OT* oral transudate

A positive correlation was established between the results of the in-house ELISA for the serum and OT samples (Spearman's correlation coefficient *r*_s_ = 0.6687, *P* < 0.0001) when all samples were studied (Fig. 4). The positive correlation improved when only Asturias dogs (negative control) and sick dogs (positive control) were investigated (Spearman's correlation coefficient r_*s*_ = 0.7479, *P* < 0.0001) and also when only Asturias seronegative dogs and high and medium seropositive dogs were studied (Spearman's correlation coefficient r_*s*_ = 0.7585, *P* < 0.0001). On the other hand, when only low seropositive dogs were investigated, the positive correlation was lower (Spearman's correlation coefficient r_*s*_ = 0.3079, *P* = 0.005).

**Fig. 5:**
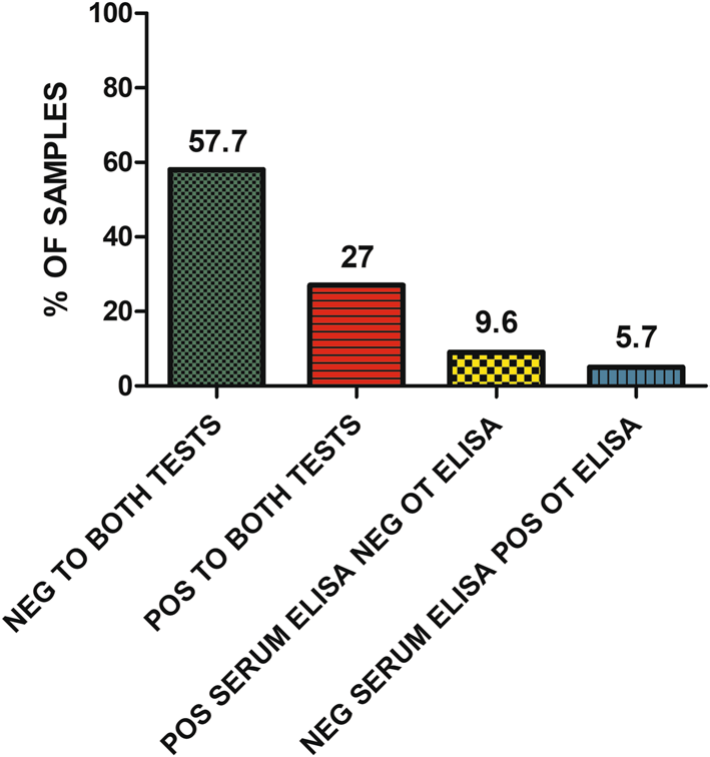
Proportion of positive and negative samples based on the results of both the serum and OT ELISAs. *Neg* Negative, *Pos* positive

Of the total of 407 dogs, 235 (57.7%) were negative by both serum and OT ELISA while 110 (27%) were positive to both tests. In contrast, there was disagreement regarding the remaining 62 dogs (15.3%). Six medium seropositive and 33 low seropositive dogs (9.6%) with a median of 55.3 EU (ranging from 35 to 288.9 EU) were negative by OT ELISA with a median of 12.4 EU (ranging from 0 to 27.2 EU) while 23 seronegative dogs (5.7%) with a median of 16.7 EU (ranging from 0.9 to 30.8 EU) were positive by OT ELISA with a median of 43.4 EU (ranging from 29.4 to 76.4 EU) (Fig. 5). The percentage agreement and Cohen’s kappa agreement between serum and OT ELISA results was substantial (0.66) when studying the whole group while it went from almost perfect to moderate depending on the classification studied (Table 2).

Comparison of the EU values for the serum and OT samples according to degrees of reactivity is shown in Table 3. When comparing the OT EU, antibody levels were found to be significantly higher in OT samples with a high or medium positive EU value for the serum ELISA than in those with a low positive serum ELISA (Kruskal–Wallis H-test: *χ*^2^ = 43.2, *df* = 2, *P* < 0.0001).

## Discussion

A quantitative in-house ELISA technique [21] was adapted in the present study to detect specific anti-*Leishmania* antibodies in OT canine samples and to assess the diagnostic performance of this ELISA. This ELISA is currently performed on serum samples to detect specific immunoglobulins as it has been proven that most dogs infected with an active disease show high levels of different isotypes of antibodies [9, 18, 37]. The presence of several types of immunoglobulins has also been studied in saliva [28]. IgA has been proven to be present in saliva as it is secreted in the salivary glands by plasma cells, and plasma-derived antibodies have been found, such as IgG and IgM [28]. Specific canine anti-*Leishmania* antibodies have also been documented in oral fluid samples by using a TR-IFMA [24, 29–31], which is a technique that has shown a broader range of detection of antibodies in serum than ELISA. These studies showed great success at discriminating between seropositive and seronegative dogs with no overlapping in terms of evaluating IgG2 [24, 29–31]. However, the authors of these studies were not successful at correctly differentiating seropositive dogs from seronegative based on IgA evaluation [24, 29–31]. These studies provided the first evidence of the potential of oral fluid for the quantification of anti-*Leishmania* IgG2 to diagnose CanL [24, 29–31]. Nonetheless, no studies have evaluated the ability to detect anti-*Leishmania* antibodies by using a quantitative in-house ELISA technique in OT samples until now. Additionally, the first study performed on oral fluid samples for the diagnosis of CanL was carried out on a very homogeneous group of dogs, using dogs with advanced clinical leishmaniosis and high antibody levels [24], while in the present study, dogs with subclinical infection and low antibody titers were also included.

In the present study, the agreement between the qualitative interpretation of serum and OT ELISA results was evaluated using two methods: (1) percent agreement and (2) agreement according to the kappa agreement statistic. The percent agreement is easy to calculate and can be interpreted directly, but it does not take into account the agreements made by chance [38]. On the other hand, Cohen’s kappa agreement statistic is a statistical value useful for assessing inter-rater or intra-rater reliability and takes into consideration the possibility of chance [38]. A Cohen’s kappa agreement of > 0.80 is needed to be able to validate a new test [38]. When Cohen’s kappa agreement was interpreted for the 407 dogs, a substantial agreement of 0.66 was found. As stated earlier in this text, this agreement is not sufficient to affirm that OT can be used to correctly differentiate between seropositive and seronegative dogs by means of an in-house ELISA. However, a high number of dogs in this study presented subclinical infection and low seropositive antibody levels, which is a likely explanation of why the agreement was lower than found in previous studies where the dog populations studied were mostly sick dogs with advanced clinical leishmaniosis [24, 31]. When Cohen’s kappa agreement was obtained only for seronegative dogs from Asturias (a low endemicity area) and for sick dogs with clinical signs and/or clinicopathological abnormalities compatible with *L. infantum* infection, an almost perfect agreement of 0.84 was obtained. The same result (0.84) was found when Cohen’s kappa agreement was obtained for seronegative dogs from Asturias and seropositive dogs with high or medium levels of antibody levels. These findings agree with those reported in previous studies [24, 31] and highlight the usefulness of detecting antibodies against *L. infantum* in OT in dogs with clinical leishmaniosis or progressing towards disease.

When the percent agreement was evaluated, an agreement of 84.8% was found. The remaining samples from 15.2% (62) dogs showed disagreements between the serum and OT ELISA. Included in these samples that disagreed, 39 were from seropositive dogs (39/62 dogs; 62.9%) that were negative by the OT ELISA. There are several reasons that could explain this disagreement in results from the OT and serum ELISA. First, there may be a lesser ability to detect seropositive dogs with a low serum antibody, as detected when comparing the Cohen’s κ agreement statistic described above. This seems to be the most plausible reason as when only seronegative dogs from Asturias and sick dogs with clinical signs and/or clinicopathological abnormalities compatible with *L. infantum* infection were studied, the percent agreement increased to 92.1%. A similar result, i.e. 92%, was obtained when the results from only seronegative dogs from Asturias and seropositive dogs with high or medium levels of antibody levels were considered. This result was to be expected as the sick group presented a higher proportion of high serum antibody levels compared to the apparently healthy group which had a higher proportion of low antibody levels. Another explanation could be a lack of homogenous OT sample collection, as even if untrained personnel can perform this procedure, it is difficult to perform correctly if the standardized protocol is not followed as described [39]. For example, if the impregnated swabs were not kept in the mouth of the dog for at least 2 min, insufficient OT could have been absorbed. As the samples in this study were collected by several veterinarians, even though a standardized protocol was recommended and agreed to, we could not confirm that all samples were always collected in a similar manner. On the other hand, of these 62 disagreements, 23 seronegative dogs (23/62 dogs; 37.1%) turned out to be positive in the OT ELISA. These results were unexpected. One possible explanation is that sand flies mainly feed on skin areas with very little hair, such as the face [15], which could lead to a local expression of parasite-specific immunoglobulins before the parasite disseminates systemically. A second possibility is that there may be an as-yet unknown cross-reactivity with another pathogen, such as oral bacteria, in some dogs with poor dental hygiene and dental disease, such as gingivitis, stomatitis and periodontal disease. Further studies on the diagnostic performance of the OT ELISA are needed to evaluate this hypothesis.

When taking locations of origin into consideration, the percent agreement was higher in Asturias (93.6%), followed by Barcelona (90%), Cádiz (88.9%) and Mallorca (79.6%). In comparison, Cohen’s kappa agreement was substantial in Barcelona (0.73), followed by Cádiz with a moderate agreement (0.56) and Mallorca, also with a moderate agreement (0.54).

Despite the OT showing a lower diagnostic value than serum according to the quantitative in-house ELISA used in this study, a good percentage of success was obtained for the OT samples. In addition, OT sample collection is easy, cheap, non-invasive and painless; consequently, OT could be of use in specific cases, such as dogs who do not have easy access to veterinary clinics, dogs that need a continued follow-up or aggressive dogs that can only be touched by its owner.

Further studies are needed to increase the reliability of the results of the present study. First, an investigation of the OT quality must be performed to confirm the correct collection of the samples before performing OT ELISA. In addition, a group of dogs with poor dental hygiene and presenting dental diseases could be added to the study population to assess the possibility of poor dental health being a factor of false positivity by OT ELISA. Also, it would be also of interest to perform a longitudinal study of those dogs that were seronegative yet tested positive by OT ELISA, as well as those dogs that tested negative for the OT ELISA yet tested positive by the serum ELISA, to describe antibody kinetics. Finally, other techniques using OT could also be developed and improved. Even ELISA as a serological test has some limitations in terms of the detection of infection as it can detect antibodies elicited by *Leishmania* vaccines in dogs [17].

The seroprevalence of canine *L. infantum* infection was around 10% [14, 33, 40] between 2011 and 2020 in Spain, which is lower than the seroprevalence detected in the present study (36.6%). In terms of specific Spanish areas, Asturias has always presented one of the lowest seroprevalence rates [14, 33, 40], usually around 1%, while the rates from Cádiz and Mallorca are usually higher than 15% [14]. These results resemble those found in the present study, with low rates in Asturias (0%) and high rates in Cádiz (16.7%) and Mallorca (35.1%). Regarding the results found in Barcelona (27.3%), a previous study performed in 27 sick and 20 clinically healthy dogs in 2006 [41] documented a 65% seroprevalence of *L. infantum* in Barcelona, but no other studies in this area have been carried out in the last decade. However, seroprevalence rates of around 13% were detected in other areas of Catalonia [33, 40]. Interestingly, the seroprevalence rates detected in this study seem to be slightly higher than those described in previous studies [14, 33, 40, 41]. This could be related to the number of sick dogs included in the Barcelona (12.7%) and Mallorca (7.4%) groups. The incidence rate of human leishmaniosis in Spain was 0.62 cases per 100,000 inhabitants between 2005 and 2017, with cases mainly distributed throughout the Mediterranean region [42]. However, asymptomatic infections are also common in humans in Spain and Mediterranean basin countries as recently reviewed elsewhere [43].

We also detected higher serological rates of *L. infantum* infection in both adult and sick dogs. A high rate should be expected in sick dogs that have been already diagnosed with leishmaniosis and still present clinical signs and/or clinicopathological abnormalities [9, 15]. In terms of age of dogs, previous studies have found that puppies (< 1 year old) have a lower rate of *L. infantum* infection than dogs aged > 1 year old [33, 40] and that the risk of *Leishmania* infection increases with increasing age [40].

## Conclusions

In conclusion, the present study demonstrates an almost perfect to moderate agreement between OT and serum samples using a quantitative in-house ELISA for *Leishmania* antibodies. These results are promising for the detection of infection in sick dogs with high antibody levels while they seem to be less optimal in apparently healthy dogs with low antibody levels. Further studies could improve OT serology and its reliability and value as a future diagnostic technique for *L. infantum* infection when compared with other diagnostic methods for CanL.

## Data Availability

The datasets used and/or analyzed during the current study are available from the corresponding author on reasonable request.

## Abbreviations

CanL: Canine leishmaniosis
ELISA: Enzyme-linked immunosorbent assay
EU: ELISA units
IFN-γ: Interferon gamma
IgA: Immunoglobin A
IgG: Immunoglobin G
IgM: Immunoglobin M
OT: Oral transudate
PBS: Phosphate buffered saline
ROS: Reactive oxygen species
SD: Standard deviation
TR-IFMA: Time-resolved immunofluorometric assay
